# Rationale and design for the Predictors of Arrhythmic and Cardiovascular Risk in End Stage Renal Disease (PACE) study

**DOI:** 10.1186/s12882-015-0050-4

**Published:** 2015-04-24

**Authors:** Rulan S Parekh, Lucy A Meoni, Bernard G Jaar, Stephen M Sozio, Tariq Shafi, Gordon F Tomaselli, Joao A Lima, Larisa G Tereshchenko, Michelle M Estrella, W H Linda Kao

**Affiliations:** 1 Department of Medicine, School of Medicine, Johns Hopkins University, Baltimore, USA; 2 Department of Epidemiology, Bloomberg School of Public Health|, Johns Hopkins University, Baltimore, USA; 3 Department of Biostatistics, Bloomberg School of Public Health, Johns Hopkins University, Baltimore, USA; 4 Welch Center for Prevention, Epidemiology, and Clinical Research, Baltimore, USA; 5 Nephrology Center of Maryland, Baltimore, USA; 6 Departments of Paediatrics and Medicine, Hospital for Sick Children, University Health Network and University of Toronto, Toronto, ON Canada; 7 Knight Cardiovascular Institute, Oregon Health & Science University, Portland, OR USA

**Keywords:** Dialysis, Hemodialysis, Mortality, Sudden death, Sudden cardiac death, Arrhythmia, End stage renal disease

## Abstract

**Background:**

Sudden cardiac death occurs commonly in the end-stage renal disease population receiving dialysis, with 25% dying of sudden cardiac death over 5 years. Despite this high risk, surprisingly few prospective studies have studied clinical- and dialysis-related risk factors for sudden cardiac death and arrhythmic precursors of sudden cardiac death in end-stage renal disease.

**Methods/Design:**

We present a brief summary of the risk factors for arrhythmias and sudden cardiac death in persons with end-stage renal disease as the rationale for the Predictors of Arrhythmic and Cardiovascular Risk in End Stage Renal Disease (PACE) study, a prospective cohort study of patients recently initiated on chronic hemodialysis, with the overall goal to understand arrhythmic and sudden cardiac death risk. Participants were screened for eligibility and excluded if they already had a pacemaker or an automatic implantable cardioverter defibrillator. We describe the study aims, design, and data collection of 574 incident hemodialysis participants from the Baltimore region in Maryland, U.S.A.. Participants were recruited from 27 hemodialysis units and underwent detailed clinical, dialysis and cardiovascular evaluation at baseline and follow-up. Cardiovascular phenotyping was conducted on nondialysis days with signal averaged electrocardiogram, echocardiogram, pulse wave velocity, ankle, brachial index, and cardiac computed tomography and angiography conducted at baseline. Participants were followed annually with study visits including electrocardiogram, pulse wave velocity, and ankle brachial index up to 4 years. A biorepository of serum, plasma, DNA, RNA, and nails were collected to study genetic and serologic factors associated with disease.

**Discussion:**

Studies of modifiable risk factors for sudden cardiac death will help set the stage for clinical trials to test therapies to prevent sudden cardiac death in this high-risk population.

## Background

Despite improvements in access to care, the dialysis procedure, and management of complications related to end-stage renal disease (ESRD), mortality in ESRD remains high [1]. Sudden cardiac deaths (SCD) are common among dialysis patients with approximately 25% of deaths in both observational studies and clinical trials [2]. Published rates of SCD among the ESRD populations vary greatly based on definitions used to define it [3]. In contrast to the general population, the incidence rate of cardiac arrest as a surrogate of SCD in ESRD is almost 50 fold greater [4]. These rates of SCD among hemodialysis patients approach similar rates in persons who experienced myocardial infarction in the general population. Despite this high-risk, few prospective studies have been conducted to define the incidence and risk factors for SCD in ESRD. Moreover, therapy including implantable defibrillators is controversial [5-7]. Herein, we focused our discussion on the main goals of the Predictors of Arrhythmic and Cardiovascular Risk in End Stage Renal Disease (PACE) study to understand clinical- and dialysis-related factors contributing to arrhythmias and SCD.

### Assessment of arrhythmic risk

Key to the pathogenesis of arrhythmias and SCD is the abnormal myocardium which is highly susceptible to abnormal ventricular conduction either spontaneously or via additional triggers. There are a number of noninvasive markers of myocardial vulnerability including electrocardiogram (ECG) changes at rest or from ischemia, and alterations in dynamic ECG parameters which can be used to risk stratify persons at risk for sudden death and represent intermediate markers of SCD risk [8,9]. Some of these ECG measures represent various mechanisms of developing life-threatening arrhythmias and include heart rate variability (HRV), ventricular late potentials (VLP), and QT interval prolongation.

HRV is defined as the variation in RR intervals and serves as a surrogate marker of autonomic dysfunction [10]. Alteration of the balance between sympathetic and parasympathetic tone by disease states can lead to depressed vagal tone and a resultant predominance of sympathetic activity, leading to tachycardia and cardiac electrical instability. In persons with chronic kidney disease, sympathetic hormone levels are 5 times greater than healthy controls [11]. Arterial hypotension during hemodialysis also leads to increased sympathetic activity. Depending on the studies, there is a wide range of estimated prevalence of abnormal HRV in dialysis from 16 to 76% [12-16]. Severe or moderately depressed HRV is more common in those receiving hemodialysis than those on peritoneal dialysis or the general population, and may also be affected by diabetes and hemodialysis treatments [12-21].

VLPs are low amplitude signals, in the microvolt range, occurring in the last few milliseconds of the QRS complex and extending into the ST segment. It is normally hidden by the much larger voltages of the QRS and ST segments and can only be detected using signal averaged ECG (SAECG) [22]. VLPs represent conduction delay within the myocardium due to fibrosis, ischemia, or other disease states. Previous studies have demonstrated VLPs as highly specific predictors of both inducible ventricular tachycardia in the general population and SCD in persons having a myocardial infarction [23]. The prevalence of VLPs in hemodialysis patients ranges from 7 to 25%, but does not consistently vary with dialysis treatment or electrolyte changes [22,24-26].

The QT interval is measured from the first deflection of the QRS complex to the end of the T wave and corrected for the heart rate using any one of a number of corrections [27]. A prolonged QT interval reflects delayed repolarization. Temporal variability of the repolarization can result in electrical instability and ventricular arrhythmias [8,28], and a prolonged QT has been shown to be associated with a higher incidence of SCD [27,29,30]. The QT interval is influenced by patterns of ventricular activation, heart rate, and autonomic tone. Electrolyte imbalances and drugs are also common causes of prolonged QTc. Prolonged QTc greater than 0.44 ms is present in 3-87% of hemodialysis patients prior to a dialysis treatment depending on population and increases to 6-99% after hemodialysis [31-40]. The QTc is longer in patients on hemodialysis than in those on peritoneal dialysis, possibly related to lower serum potassium and calcium levels [41]. Calcium primarily affects the QT interval and may increase or decrease depending on dialysis bath and dialysis treatments [36]. Elevated QT variability index has also been described in 47% of persons with chronic kidney disease and is associated with diabetes and baseline coronary artery disease [42].

### Assessment of vulnerable myocardium

Left ventricular hypertrophy (LVH) and structural heart disease are strongly associated with the development of SCD in the general population [43,44]. Similarly, in the dialysis population, greater left ventricular (LV) mass index is an independent predictor of SCD [45]. LVH is present in 40 to 74% of patients with ESRD [46,47] and could reduce coronary blood flow and increase oxygen consumption of the myocardium. This imbalance in coronary blood flow and myocardial oxygen requirement can subsequently lead to ischemia, prolongation of the QTc interval, and arrhythmias [39,48-51]. Worsening LVH is also associated with new onset heart failure [52], which occurs in 7% of the ESRD population annually [4,53]. Though traditional baseline risk factors for atherosclerotic cardiovascular disease are associated with a higher risk of SCD, additional measures of subclinical cardiomyopathy and cardiovascular disease that are more common in dialysis patients, such as coronary and vascular calcification, and vascular stiffness, need to be studied with respect to arrhythmic risk [54].

### Dialysis-related risk factors associated with SCD and arrhythmias

With the typical conventional regimen of thrice weekly hemodialysis, patients are subjected to a particularly unphysiological state that includes elevated serum potassium and magnesium before dialysis and low after dialysis, low calcium or high calcium-phosphorus product, acidosis, hypertension, and fluid overload. All of these conditions have been associated with lethal arrhythmias and SCD in the general population, especially in those with prevalent cardiovascular disease [55-58]. Hemodialysis is not administered for 2 consecutive days each week, resulting in even more significant fluid gain, hypertension and elevated potassium. Mortality among hemodialysis patients is also more frequent on Mondays and Tuesdays after the 2 days off dialysis suggesting that, indeed, the unphysiologic milieu leads to lethal arrhythmias and SCD [59]. In fact, in-unit cardiac arrest is also more common on Mondays and more likely when the patient is dialyzed against a 0 or 1.0 mEq/L potassium dialysate (17.1 vs. 8.8%) [60].

Arrhythmias can occur at initiation of dialysis and also toward the end of the dialysis session, probably due to fluctuations in electrolytes and fluid [11]. Exaggerated sympathetic responses also occur at higher rates of ultrafiltration [17]. Concentrations of potassium and calcium, important in regulation of electrical conduction in the heart, are adjusted in the dialysis solution (or dialysis bath) on an individual patient basis, perhaps increasing the arrhythmic risk [60]. Low calcium dialysate is now commonly used because of increasing total body calcium burden in ESRD. A recent study has demonstrated the relationship between lower calcium dialysate and higher SCD relationship between using a case- control design and requires further confirmation [61].

Additional factors during dialysis therapy such as hypotension and increased ultrafiltration lead to diminished coronary and cerebral perfusion which, in turn, promotes arrhythmias. Such episodes of hypotension are independently associated with in-unit cardiac arrest [60]. In persons with intradialytic hypotension, autonomic control of the heart is impaired [18] and typically associated with impaired LV diastolic function [18,62]. Prospective studies are needed to assess the relative roles of intradialytic hypotension and ventricular function, and the risk for arrhythmias and SCD. To that end, the Predictors of Arrhythmic and Cardiovascular Risk in End Stage Renal Disease study was initiated.

## Methods

### Study design of the predictors of arrhythmic and cardiovascular risk in end stage renal disease (PACE) study

**Figure 1:**
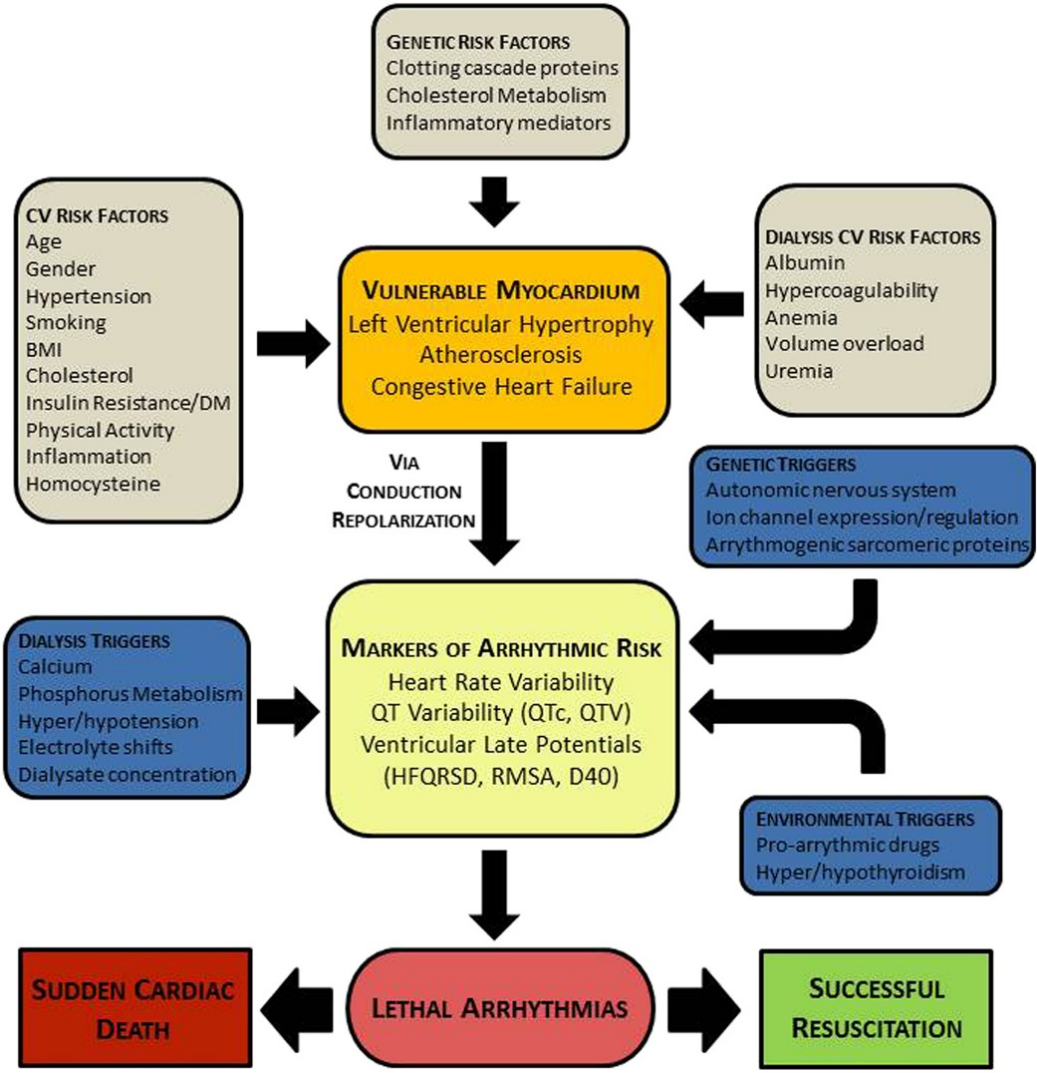
Conceptual model of the predictors of arrhythmic and cardiovascular risk in end stage renal disease (PACE) study.

The overall objective of the study was to determine risk factors (cardiovascular, dialysis-related, environmental and genetic) associated with disordered cardiac autonomic regulation and ventricular conduction, as well as with incidence of SCD, in ESRD patients treated with hemodialysis. The study was organized around the following aims and followed the conceptual model based on the understanding of risk factors leading to SCD (Figure 1).

1. Establish a cohort of incident hemodialysis patients and describe the incidence of SCD
2. Determine the association of putative risk factors with disordered cardiac autonomic regulation and ventricular conduction in both cross-sectional and prospective analyses
3. Determine the association of disordered cardiac autonomic regulation and ventricular conduction and other putative risk factors with incidence of SCD over follow-up.

### Study overview and inclusion criteria

This prospective cohort study recruited persons with incident ESRD in Baltimore and the surrounding area from 25 free-standing outpatient dialysis units (DaVita and Medstar) and 2 hospital-based outpatient units (MedStar). Incident dialysis was defined as less than 6 months on regular outpatient thrice weekly hemodialysis.

The participants were screened in the dialysis unit to determine eligibility and, if eligible, were consented. The inclusion criteria for participation were as follows: (1) incident hemodialysis therapy defined as starting within the 6 months before enrollment; (2) signed informed consent; (3) age 18 years or older; (4) English speaking; and (5) ability to tolerate the imaging and ECG procedures and complete questionnaires. The criteria for exclusion were as follows: (1) home hemodialysis or peritoneal dialysis patients; (2) patients in hospice or skilled nursing facility or prison; (3) persons with cancer other than nonmelanoma skin cancer; (4) presence of a pacemaker; (5) presence of an automatic implantable cardioverter defibrillator; (6) pregnant or nursing mothers (pregnancy test done prior to imaging); and (7) health conditions that interfere with study participation (such as substance abuse, dementia, or psychotic illness).

The consented participants were interviewed in the dialysis unit and brought to the Johns Hopkins Institute for Clinical and Translational Research (ICTR) clinic located at Johns Hopkins Hospital for a clinic visit on a nondialysis day. These dialysis unit and clinic visits were performed annually for up to 4 years. Participants were also followed semi-annually with a phone call to assess recent hospitalizations. In addition, at every 4- and 8-month interval, the participants were called by trained dieticians to reassess their dietary 24-hour recall. Lastly, every 6 months, the coordinators contacted the clinic to determine dialysis discontinuation, hospitalizations, and vital status information.

### Study procedures

Briefly, we conducted detailed questionnaires in the dialysis unit and at the clinic visits at baseline and at annual study visits (see Table 1 for schedule and questionnaires). Detailed questions on socio-demographic information, education, employment, family history, medications, medical history, female reproductive history, quality of life, recreational drug use, depressive symptoms, cognitive function, frailty, physical activity, dietary history, smoking, alcohol history, and transplant evaluation were included.

**Table 1:** **Schedule of measurements and activities by visit for the PACE study**

	Baseline visit	Follow-up (FU) Visits and Telephone (T) Contacts									
	EV	BV	T4	T6	T8	FU12	T16	T18	T20	FU24	Up to FU48
**Interviewer/Questionnaires** ^**§**^											
Informed consent	●										
Contact information	●			●		●		●		●	●
Sociodemographic	●										
Education and employment		●				●				●	●
Vascular access		●				●				●	●
Residual kidney function		●				●				●	●
Dialysis prescription		●				●				●	●
Dialysis adherence		●				●				●	●
Pre-dialysis education		●				●				●	●
Medical history	●			●		●		●		●	●
Family history of CVD, SCD	●										
Female reproductive history		●				●				●	●
Health, behaviour, social history	●					●				●	●
Access to transplant	●					●				●	●
KDQOL-36		●				●				●	●
Symptomatic CVD (Rose, KCQ)		●				●				●	●
Physical activity		●				●				●	●
Dietary questions & 24-hr recall		●	●		●	●	●		●	●	●
Literacy (WRAT4)		●									
Cognition (Trails A&B, 3MS)		●				●				●	●
Depression (PHQ9)		●				●				●	●
Oral medications		●				●				●	●
**Examination**											
Height, weight, BMI, WHR		●				●				●	●
Blood pressures		●				●				●	●
Detailed amputation evaluation		●				●				●	●
Frailty assessment		●				●				●	●
**Procedures**											
CT calcium and angiography		●									
Echocardiography		●									
Pulse wave velocity		●				●				●	●
12 lead & signal averaged ECG		●				●				●	●
4 Limb ankle brachial		●				●				●	●
**Blood tests (fasting)**											
Ionized calcium, magnesium		●				●				●	●
Repository (DNA, RNA, blood, nails)		●				●				●	●
**Clinical**											
Baseline charlson co-morbidity	●										
CMS-2728 medical evidence	●										
Hospitalizations	**Throughout the entire study period**										
CMS-2746 death notification^**‡**^	**Collect as needed**										
**Dialysis routine bloods (monthly)**	**Throughout the entire study period**										
Glucose, albumin											
Complete blood count											
Electrolytes (Na, K, Mg, CO2, Ca, Phos)*											
Intact PTH*											
Fe, TIBC, Ferritin, Aluminum*											
URR, spKt/V, nPCR*											
**Dialysis treatments (weekly)** ^**‡‡**^	**Throughout the entire study period**										
Dry weight											
Dialysis bath (Ca, K, HCO_3_)*											
Prescription (duration, frequency, dialyzer)											
Pre and post weights											
Pre and post blood pressures (BP)											
Dialysis flow rate											
Blood flow rate											
Intravenous medications											

^**§**^**KCQ = Kansas City Questionnaire;** KDQOL-36 = Kidney Disease Quality of Life 36 Item Instrument; WRAT4 = Wide Range Achievement Test 4^th^ Edition; Trails A&B; 3MS = Modified Mini-Mental Status Exam; PHQ9 = Patient Health Questionnaire 9; ¶BMI = Body Mass Index; WHR = Waist Hip Ratio; Blood Pressure is collected as 3 seated Blood Pressures; ‡Collected as needed; *Na- sodium K- potassium; Ca- calcium; Phos- phosphorus; HCO_3_- bicarbonate; PTH- parathyroid hormone level; Fe- iron; TIBC- total iron binding capacity; URR- urea reduction ratio; nPCR- nitrogen protein catabolic rate; ‡‡Dialysis Machine Derived Parameters: BP-pre, BP-post, Lowest BP, Weight-pre, Weight-post, Intradialytic Weight Gain, & Average Blood Flow Rate.

For the clinic visits, participants came to the Johns Hopkins ICTR clinic for a physical examination, cardiac evaluation, cognitive testing, and frailty assessment. All participants were instructed to fast for at least 8 hours prior to the study visit. The first study visit was always done at the earliest appointment in the morning, and participants were encouraged to not engage in activity as they were fasting prior to the visit. All study evaluations were conducted on nondialysis days to ensure uniformity across the study population. The visits included a physical examination (anthropometry and waist to hip ratio), three resting blood pressures using an oscillometric machine, frailty assessment (grip strength and timed walking measurement), and cardiovascular assessments (four-limb blood pressure, if indicated, for ankle brachial index, pulse wave analysis and velocity, standard 12-lead ECG, and two 5- and 2-minute digital signal averaged ECG recordings). Participants were asked to bring in their medications and all outpatient medications were recorded. Additionally, computed tomography [CT] scan for coronary and valvular calcium and angiography (if not contraindicated), and echocardiogram were conducted at baseline only. Biological specimens were collected at the time of each study visit for the biorepository. Measurement of ionized calcium and serum magnesium were done at each study visit.

### Baseline and annual data collection

Data collected from questionnaires have primarily used standardized, validated, and reliable questionnaires similar to the Chronic Renal Insufficiency Cohort (CRIC) Study [63,64]. Race was self-reported. Physical activity was determined using the Minnesota Activity Index [65]. Dietary intake data using the 24-hour food frequency questionnaire/assessment were collected and analyzed using Nutrition Data System for Research software versions 2009–2013 developed by the Nutrition Coordinating Center (NCC), University of Minnesota, Minneapolis, MN. The Nutrition Data System for Research provides a complete nutrient profile for all foods in the database.

Additional data prior to or at dialysis initiation were collected from medical records and the Center for Medicaid and Medicare Services (CMS) form-2728. Electronic dialysis records for all treatments during the study period for all participants were also provided by the dialysis providers, DaVita and MedStar, with. These data included laboratory, treatment, medications, and dialysis prescription values.

### Cardiovascular evaluation

#### Electrocardiogram (ECG) recording and analysis

We conducted ECG recording at baseline and annual follow-up visits. We defined specific ECG parameters in Table 2. A standard 12-lead ECG was recorded at rest in supine position using the Marquette MAC 5500 HD ECG system (GE Medical Systems, Milwaukee, WI). High resolution (1000 Hz). Orthogonal Frank XYZ ECG was recorded using the Norav 1200 M PC ECG machine (Norav Medical Ltd, Thornhill, ON, Canada) at rest in the supine position for at least 5 minutes. Only sinus beats were included in the analysis. Subjects with serious cardiac arrhythmias defined as ventricular tachycardia, ventricular fibrillation, and atrial fibrillation/flutter were captured, if detected, based on standard criteria [9]. Atrial fibrillation/flutter, as well as premature atrial and ventricular contractions with the first subsequent beat were excluded from subsequent analyses. Fiducial points were automatically detected on each ECG lead and verified by investigators as previously described [66]. Bazett (QT_B_) [67], Fridericia (QT_F_) [68], and individualized (QT_i_) [69] approaches for QT correction were applied. Both time domain and frequency domain HRV analyses were performed as recommended by the European Society of Cardiology/North American Society of Pacing and Electrophysiology (ESC/NASPE) Task Force, as well as the American College of Cardiology (ACC)/ American Heart Association (AHA) guidelines for analysis of short-term recordings [9]. Robust automated template matching techniques were used for QT variability analyses [70,71].

**Table 2:** **Cardiovascular assessment of PACE participants**

Imaging study	Procedure	Measures collected	Definitions/Analyses
**Signal-Averaged Electrocardiogram (SAECG), orthogonal ECG and 12 Lead ECG**	Recorded using standard 12 and Frank orthogonal XYZ leads during a minimum of 5 minutes at rest with a 1000 Hz sampling frequency and high-pass filter 0.05 Hz and low pass filter 350 Hz.	12 Lead ECG: Heart rate rhythm	Sinus rhythm, tachy/bradycardia
PC ECG machine (Norav Medical Ltd, Thornhill, ON, Canada) and Marquette ECG* (GE Medical Systems, Milwaukee, WI)		Signal Averaged ECG: Ventricular late potentials	Ventricular late potentials considered positive with 2 or more criteria: (1) fQRSd >114 ms, (2) LAS-40 > 38 ms, and (3) RMS-40 < 20 μV, where fQRSd is total filtered QRS duration; LAS-40 is duration of the low-amplitude signals (<40 mV) in the terminal portion; and RMS-40 is root mean square voltage of the last 40 ms.
		Corrected QT interval QT variability index (QTVI)	QTc > 0.44 QTVI > −0.5
		Heart Rate Variability	SDNN < 50 ms HRV Index < 15 LF/HF > 1.5
**Multidetector Computed Tomography (CT) and Angiography (CTA)**	Prospective electrocardiographic gating with gantry rotation times 350–400 msec. Scans triggered by electrocardiography signal at 70-80% RR interval, near the end of diastole, and before atrial contractions, to minimize the effect of cardiac motion. Conducted with IV visipaque contrast if not contraindicated and metoprolol if heart rate greater than 70 beat per minute.	Left main (LM) Left anterior descending (LAD) Left anterior descending (LAD) Left circumflex (LCX) Right coronary artery (RCA)	Coronary vessel stenosis defined as significant narrowing (50% or more diameter reduction) of the lumen.
*Toshiba Aquilon 32 (Toshiba, Japan)*		Coronary calcium score Valvular calcium	Coronary calcium determined using Agatston score. Valvular calcium is measured.
**Echocardiogram**	Conducted in a reclined position with 4 chamber views and M mode echocardiography to determine left ventricular and atrial dimensions	Ejection fraction (EF)	EF = (EDV-ESV / EDV) x100%
*Toshiba Artida, (Toshiba, Japan)*		Aortic root pulmonary arterial pressure	Aortic root normal parameters = 0.6-1.1Cm tricuspid regurgitation pressures
		LV mass index*	LV mass = 0.8 (1.04 ([LVIDD + PWTD + IVSTD]^3^- [LVIDD]^3^)) + 0.6 g
**Pulse wave velocity**	Measures are performed supine after 5 minutes of rest using the right carotid and right femoral arteries. The operator captures 10 seconds of stable waveform and repeats the sequence using the femoral artery. The computer generated aortic PWV with a standard deviation is reviewed.	Carotid-femoral pulse wave velocity	
*Sphygmocor PVx system (Atcor Medical, Australia)*			
**Pulse wave analysis**	Measures are performed supine after 5 minutes of rest using the right radial artery (left radial artery if right access is dialysis access present). The operator captures 10 seconds of stable waveform. The computer generated quality indices and operator index are reviewed.	Systolic central aortic pressure Diastolic central aortic pressure Mean central aortic pressure aortic Augmentation index	
*Sphygmocor PVx system (Atcor Medical, Australia)*			
**Ankle Brachial Index (ABI)**	Measures are performed after 5 minutes of rest in a supine position and assessed by measuring blood pressure bilaterally in the brachial, dorsalis pedis and posterior tibial arteries.	Right brachial pressure right posterior tibial artery (PTA) right dorsalis pedis artery (DPA) left brachial pressure left posterior tibial artery (PTA) left dorsalis pedis artery (DPA)	Right ABI = Higher of the right ankle pressures (PT or DP)/ Higher arm pressure (right or left) Left ABI = Higher of the left ankle pressures (PT or DP)/ Higher arm pressure (right or left)
*Doppler probe*			

*by Devereux formula; LVIDD = Left Ventricular Internal Diameter in Diastole, PWTD = Posterior Wall Thickness in Diastole, IVSTD = Interventricular Septum Thickness in Diastole.

Additionally, SAECG was obtained with a MAC 5500 HD ECG system (GE Medical Systems, Milwaukee, WI), with bandpass filtering of 40–250 Hz, and averaging of 350 QRS complexes. The following SAECG characteristics of the filtered QRS were evaluated [72,73]: (1) total duration (fQRSd), (2) duration of the low-amplitude signals (<40 mV) in the terminal portion (LAS40), and (3) root-mean-square voltage of the last 40 ms (RMS40). VLPs were considered positive when ≥2 of the following criteria were fulfilled: (1) fQRSd >114 ms, (2) LAS40 > 38 ms, and (3) RMS40 < 20 μV.

All ECG measures are analyzed as continuous variables or clinical categories based on standardized methods [9,55], and are studied as potential effect modifiers [74].

#### Subclinical measures of myocardial substrate

All subclinical CVD measures used standardized protocols similar to the CRIC Study (Table 2) [63,64] with corresponding quality control procedures. We conducted the CT at baseline on each participant to determine coronary artery calcium and arterial stenoses. Agatston coronary calcification scores, volume, volumetric scores, and mass for the four main coronary arteries and cardiac valves were calculated. Aortic or mitral valvular calcification was also defined as present or not. Coronary artery stenosis was defined as significant narrowing (50% or more diameter reduction) of the lumen of the four main coronary branches: left main, left anterior descending, left circum-flex, and right coronary artery, including side branches according to the AHA classification [75]. The effective radiation dose estimation for the entire study that includes CT angiography and calcium measurement was approximately 5–7 mSv (~0.7 rem). Prior to the study, a beta-blocker, metoprolol, was given orally to bring the heart rate to less than 65 beats per minute. Based on protocol, trained cardiologists and radiologists independently read all CT images to assess for both cardiac and non-cardiac pathologies and submitted a separate report of these findings within a week [76].

Echocardiograms were conducted by three trained echocardiographers at baseline on each participant to determine LV stroke volume, LV ejection fraction, and LV mass index with four chamber views and standard calculations for LV mass. The M-mode by the parasternal short axis view was used to estimate LV mass, as the long axis view can result in improper alignment and overestimate LV dimensions and mass [77]. Additionally, we collected pulmonary artery pressures based on flow at the tricuspid valve. This was not always possible based on body habitus and lack of regurgitation evident at the valve. All cardiovascular studies were centrally read at the Johns Hopkins Cardiovascular Laboratory.

Aortic pulse wave velocity (PWV) measures were performed supine after at least 5 minutes of rest using the right carotid and right femoral arteries by four trained research staff [78]. The operator captured 10 seconds of stable carotid waveform and repeated the sequence using the femoral artery. After the second waveform was captured, the computer generated an estimate of aortic PWV with a standard deviation. If the standard deviation was more than 15% of the PWV value, the study was repeated. Pulse wave analysis was also measured using tonometry of the right radial artery (and left if arteriovenous fistula was present). Quality control was assessed in real-time using quality indices and operator index for each waveform generated. Additional internal quality control included review of study procedures by operator every 6 months to ensure adherence to the protocol.

Ankle brachial index (ABI) was performed with the patient resting for 5–10 minutes in a supine position and assessed by measuring blood pressures with a Doppler probe bilaterally in the brachial, dorsalis pedis, and posterior tibial arteries. The right and left ankle–brachial index values were determined by dividing the higher ankle pressure in each leg by the higher arm pressure.

### Adjudicated baseline comorbidities

Baseline comorbidity was assessed by the Charlson comorbidity index and was adjudicated by the PACE Endpoint Committee with two independent reviews and a third final review. If a a consensus was not reached, other members of the committee reviewed the chart and a majority vote of the committee determined the final cormorbidity. Assigned cause of ESRD was obtained from medical record review, kidney biopsy records if provided, and CMS-2728.

### Adjudicated clinical cardiovascular events and outcomes

All clinical events from hospitalizations and emergency room visits during follow-up used adjudication protocols similar to the CRIC Study [63,64], except for arrhythmias by ECG which were assessed annually during study visits. All deaths are currently being adjudicated by the PACE Endpoint Committee with two independent reviews of deaths. If there is not a consensus, a third member of the committee review the chart and a majority vote of the committee determines the final cause of the death.

#### Cardiovascular events

We also obtained discharge summaries for all hospitalizations as reported by participants or dialysis unit. In addition, surveillance of hospitals commonly frequented by participants was conducted to minimize missed emergency room visits, hospitalizations, or deaths. Also, linkage to the United States Renal Data Systems (USRDS) in the future will allow for additional data capture on healthcare utilization.

We define composite outcomes of cardiovascular disease (Table 3). Atherosclerotic disease is classified by standardized definitions for myocardial infarction, coronary revascularization procedures, stroke, carotid endarterectomy, congestive heart failure, and peripheral vascular disease by amputation or peripheral surgical/ percutaneous. Clinical or symptomatic arrhythmic events include treated atrial fibrillation/atrial flutter, heart block or bradycardias, ventricular fibrillation or tachycardia, and additional procedures for a pacemaker or defibrillator or electrocardioversion or ablation. Composite of arrhythmic disease is: (1) atrial arrhythmias including atrial fibrillation by annual ECGs and new arrhythmic events such as treated atrial fibrillation by electrocardioversion or ablation or rate control by pharmacological intervention, and death related to atrial fibrillation [79,80]; and (2) ventricular-related arrhythmias including ventricular tachycardia by annual ECGs, SCD, hospitalized arrhythmias, and placement of implantable defibrillators [81].

**Table 3:** **Adjudicated hospitalization events in the PACE study**

Event			Adjudicated based on following clinical criteria
**Cardiovascular disease (CVD)**			
**Myocardial infarction**	Clinical symptoms		Jaw pain or chest Pain
	ECG markers		Major Q wave
			ST elevation with or without Q wave
	Cardiac biomarkers		Troponin I
			CK-MB or CK total
**Congestive heart failure/Volume overload**	Clinical symptoms		Dyspnea on exertion or rest
			Pulmonary edema or pulmonary congestion
	Radiographic evidence		Inspiratory crackles
			S3 gallop
	Physical exam		Jugular venous distension
			Peripheral edema
**Procedures**	Atherosclerotic CVD (ASCVD)		Percutaneous transluminal coronary angioplasty (PTCA)
			Coronary artery bypass graft (CABG)
			Carotid endarterectomy
			Abdominal aortic aneurysm repair
	Peripheral vascular disease (PVD)		Peripheral angioplasty
			Peripheral bypass
			Limb amputation
**Cerebrovascular Accident (CVA) clinical and supportive CT and MRI evidence**	Large artery stenosis		>50% stenosis of major artery
			Cerebral cortical impairment
			Brainstem/cerebellar dysfunction
	Cardioembolism		
	Small-artery occlusion (lacunar)		Clinical lacunar syndromes
			No evidence of cortical dysfunction
	Other etiology		Nonatherosclerotic vasculopathies
			Hypercoagulable states or hematologic disorders
	Undetermined etiology		
**Systemic infections**	Sepsis	Central Nervous system	Access -related
	Respiratory/GI tract	Fungal	Skin
	Peritonitis	Septic Arthritis	Osteomyelitis
**Fractures**	All		Traumatic
	Hip/ Vertebral compression		Traumatic

#### Mortality

From our regular contact with the dialysis unit, we have been informed of a participant’s death. Once notified of a participant’s death, we obtained the CMS death notification form (CMS-2746). We also interviewed the next of kin to determine when the participant was last seen prior to death or the last hospitalization and whether any symptoms preceded the event. All records from the hospitalization or emergency room visit were also obtained. To ensure that death ascertainment is complete, we searched the National Death Index annually for persons lost to follow-up [82].

#### Sudden cardiac death

We used similar criteria for death classification adapted from the HEMO and CRIC studies [83]. SCD was defined as a sudden pulseless condition (collapse or syncope) presumed to be due to an arrhythmia occurring out of the hospital or in the emergency room in an otherwise stable individual. In case of an unwitnessed event, there was an evidence that the patient was seen in a stable condition within the 24 hours preceding the event (or since the last dialysis session). All events that occurred during a hospitalization, or in nursing home, or hospice were not classified as SCD. Deaths attributed to coronary artery disease as similarly defined in the Atherosclerosis Risk in Communities (ARIC) Study [84] if the patients (1) have had a definite myocardial infarction within 4 weeks of death, or (2) have had chest pain within 72 hours of death in cases of out-of-hospital death or cardiac pain in cases of in-hospital death, or (3) history of chronic ischemic heart disease such as myocardial infarction, coronary insufficiency, or angina pectoris, or (4) the underlying cause of death in the death certificate included ICD-10 code I20, I21, I22, I23, I24, I25, I46, I51.6, I51.9, R99, J96, if there was no evidence of a non-atherosclerotic or non-cardiac atherosclerotic process that was the probable cause of death.

### Biorepository

A biorepository consists of blood and toenail specimens collected at baseline and annually therafter. The biological collections include DNA, RNA, serum, plasma, whole blood, and buffy coat. Specimens were aliquoted and stored at −80°C. We also used quality control samples to assess for laboratory variation with any testing.

### Ethics and funding sources

The protocol described is approved by the Johns Hopkins School of Medicine Institutional Review Board and MedStar Institutional Review Board. The study was supported by the grant R01DK72367 from the National Institute of Diabetes and Digestive and Kidney Diseases (NIDDK) of the National Institutes of Health (NIH). Additional support has been provided through the Doris Duke Foundation and the National Kidney Foundation of Maryland. The clinical study visit was supported by funding from UL1 RR 025005 from the National Center for Research Resources (NCRR), a component of the NIH, and NIH Roadmap for Medical Research.

### Clinical characteristics of the PACE study population

We screened a total of 1736 individuals identified as incident dialysis patients by dialysis staff of which, 943 (54.3%) met eligibility criteria. Those with either a pacemaker (n = 89) or an automatic implanatable cardioverter defibrillator (n = 70) at time of screening were ineligible to participate in the study in addition to other reasons such as nursing home residents, inability to consent, history of recent cancer and history of peritoneal dialysis or transplantation. A total of 574 participants (61% of 943) were consented into the study with 402 completing the baseline cardiac evaluation. The median follow-up time was 1.78 years (range 0–5.43) with 52 participants who underwent kidney transplantation, 25 who transferred to peritoneal dialysis, and 106 who died as of July 31, 2014.

The PACE study population is predominantly younger and comprised of a larger proportion of African Americans than described in the national Comprehensive Dialysis Study (CDS) or the USRDS (Table 4) [1,85]. In Table 5, the baseline demographic and clinical characteristics are shown for the study population enrolled. The baseline characteristics of the cardiac measures, laboratory tests and medications are also provided for those who have completed the initial study visit with the detailed cardiac evaluation.

**Table 4:** **PACE study population and comparison to incident US dialysis cohorts**

Dialysis study	PACE	CDS*	USRDS*
Time period	2009-2012	2005-2007	2008
**Incident population, n**	574	1646	110,175
**African American**	66%	28%	28.8%
**Mean age, yrs**	56	60	62.8
**Younger age <65 years**	73%	61.8%	52.8%
**Diabetes**	53%	52.6%	44.9%
**Mean BMI, kg/m** ^**2**^	29.3	29.8	28.4
**Male**	54%	55.1%	57.6%
**Nutritional assessment, n**	402	361	None
**Follow-up**	Annual in-person clinical evaluations semi-annual phone interviews	Passive	Passive
**Cardiac evaluation**	Signal averaged ECG	None	None
	Echocardiogram		
	Cardiac CT calcium and angiography		
	Pulse wave velocity		
	Ankle brachial index		
**Hospitalizations/Mortality**	Adjudicated	Passive	Passive
**Baseline specimen collection**	402	269	None
**Follow-up specimen collection**	Yes	None	None

*CDS-Comprehensive Dialysis Study 1646 completed phone interview; USRDS- United States Renal Data Systems.

**Table 5:** **Baseline demographic and clinical characteristics of all enrolled PACE participants (n = 574) and completed cardiovascular study visit (n = 402)**

Characteristics	All PACE participants	Completed cardiovascular visit*
**Demographic**		
Male, n (%)	319 (56)	233 (59)
African-American, n (%)	397 (69)	288 (72)
Age in years, mean ± SD	56 (13.5)	55 (13.2)
Education, % graduated high school	62	248 (63)
Employment, % employed	12	47 (12)
Marital status, % married	31	29
**Dialysis characteristics**		
Three times a week dialysis, n (%)	566 (98.6)	397 (98.8)
Three to four hour dialysis session, n (%)	505 (88.0)	353 (87.8)
Polyflux membrane, n (%)	446 (77.7)	306 (86.4)
Arteriovenous fistula access, n (%)	163 (28.7)	122 (30.3)
**Self reported**		
Smoking, % ever smoker	59	60
Body mass index kg/m^2^, mean ± SD	29.4 (7.9)	29.3 (7.8)
CVD, % diagnosed	44	45
CHF, % diagnosed	23	25
Diabetes, % diagnosed	54	55
**Cardiovascular study visit**		
Systolic blood pressure mmHg, mean ± SD	n/a	137 (25)
Diastolic blood pressure mmHg, mean ± SD	n/a	75 (15)
Waist to hip ratio	n/a	0.95 (0.08)
Frailty, % diagnosed	n/a	40
Average literacy, mean	n/a	Grade 8
Cognitively impaired, % diagnosed	n/a	14
Depression, % diagnosed	n/a	17
**Medications use**		
Betablocker, %	n/a	58
ACEI/ARB, %	n/a	44
Calcium channel blocker, %	n/a	60
Statins, %	n/a	52
**Intradialytic labs**		
Ionized calcium mean ± SD. mmol/L	n/a	1.15 (0.07)
Magnesium mean ± SD, mg/dL	n/a	1.76 (0.24)

*Cardiovascular study visits were conducted at the Institute for Clinical and Translational Research and the Cardiology Research Laboratory.

#### Data safety monitoring board

We had an independent Data Safety Monitoring Committee with three physicians in nephrology and cardiology who reviewed any adverse events related to study procedures twice a year. The Committee reviewed events that occurred during the study visits or from the study’s cardiac imaging procedures. Clinical readings of the echocardiogram, CT angiography, and CT chest over-reads were required even though they were obtained for clinical research, as the discovery of incidental findings may have necessitated the timely reporting of critical results to the primary nephrologists, dialysis facilities, primary care physicians, and participants [76]. Depending on the findings, we had instituted a protocol to report urgent findings that required intervention to the nephrologist and participant.

## Discussion

The PACE Study is unique compared to other dialysis cohorts, as all participants are recruited within 6 months of initiating chronic hemodilaysis. Ethnic disparities are evident in survival on dialysis, and cardiovascular risk among African Americans with ESRD has not been well-studied. Morever, confounding by age-related disease may lead to biased results. The PACE cohort is enriched with African American participants and younger adults; therefore, it is well poised to evaluate these noted ethnic disparities and confounding. Additionally, the cohort has comprehensive and detailed baseline cardiovascular phenotyping and longitudinal electronic data for all outpatient dialysis treatments. The PACE study has also ascertained the important clinical endpoints of hospitalizations and adjudicated deaths. The biorepository of collected specimens at baseline and follow-up will allow future assessment of serologic or genetic markers associated with SCD or other outcomes.

Recruiting dialysis participants for both cohort studies and trials have often resulted in lower participation, the need to extend recruitment time, or inclusion of prevalent patients to achieve targets. Patients have significant morbidity and mortality especially in the first 3 months on dialysis, hence the definition of chronic dialysis is defined as dialysis longer than 3 months [1]. In recruiting the PACE study population, many participants had hospitalizations or various clinical visits (e.g. vascular access evaluation) that prevented follow-up with the study visit for cardiovascular evaluation. Nonetheless, recruitment of participants at dialysis initiation is imperative as studying prevalent patients brings an inherent survival bias to studies on cardiovascular risk. In order to recruit our dialysis cohort, we collaborated with outpatient dialysis units throughout the Baltimore region. This collaborative network and support from both community and academic nephrologists was vital to the development of the PACE cohort and longitudinal follow-up. We developed a number of strategies to improve study participation with paid transportation to and from the study visit, phone call reminders, and renumeration for each study visit attended.

Noninvasive cardiovascular imaging in studies among dialysis patients is needed to understand surrogate outcomes of cardiovascular disease and potentially develop measures to be used in the clinical setting. Conducting cardiovascular studies such as CT angiography, however, requires more careful planning, as imaging requires medication to lower heart rate prior to studies, use of intravenous contrast, need for appropriate screening for contrast reactions, concerns of preserving residual kidney function, consideration of radiation exposure, and lastly, appropriate protocols for reporting urgent clinical and incidental findings. These hidden burdens to the participant and study team require significant planning and follow-up not typical in most observational studies. Moreover, reporting of incidental findings to both participants and care providers adds significant responsibility to nephrologist and study team for reporting findings in a time sensitive manner, as well as potentially increasing the need for further imaging and work-up and increased worry to the participant. Requirements by ethics boards for vigilant reporting of incidentalomas does not take into account the lack of a actionable plans for most findings and the concerns of participants of unnecessary testing [76].

The overall rate of SCD in the U.S. dialysis population is extremely high but understudied, thus limiting our understanding of the pathogenesis leading to SCD. Studies such as PACE are needed to determine the incidence of arrhythmias and SCD in persons on hemodialysis and the association with cardiovascular and dialysis-related risk factors. The results of the studies will provide essential information to ultimately prevent fatal arrhythmias and prolong life.

## Abbreviations

ESRD: End-stage renal disease
SCD: Sudden cardiac deaths
PACE: Predictors of Arrhythmic and Cardiovascular Risk in End Stage Renal Disease Study
ECG: Electrocardiogram
HRV: Heart rate variability
VLP: Ventricular late potentials
SAECG: Signal averaged electrocardiogram
LVH: Left ventricular hypertrophy
ICTR: Institute for Clinical and Translational Research
CT: Computed tomography
CRIC: Chronic Renal Insufficiency Cohort Study
NCC: Nutrition Coordinating Center
CMS: Center for Medicaid and Medicare Services
ESC/NASPE: European Society of Cardiology/North American Society of Pacing and Electrophysiology
ACC: American Collect of Cardiology
AHA: American Heart Association
PWV: Pulse wave velocity
ABI: Ankle brachial index
USRDS: United States Renal Data Systems
ARIC: Atherosclerosis Risk in Communities
NIDDK: National Institute of Diabetes and Digestive and Kidney Diseases
NIH: National Institutes of Health
NCRR: National Center for Research Resources
CDS: Comprehensive Dialysis Study
